# Air pollution macro-regions identification using machine learning and spatio-temporal analysis

**DOI:** 10.1371/journal.pone.0340191

**Published:** 2026-01-12

**Authors:** Tymoteusz Morawiec, Mateusz Zareba, Tomasz Danek, Monika Chuchro

**Affiliations:** Department of Geoinformatics and Applied Computer Science, Faculty of Geology, Geophysics and Environmental Protection, AGH University of Krakow, Krakow, Malopolska, Poland; Military University of Technology Faculty of Civil Engineering and Geodesy: Wojskowa Akademia Techniczna im Jaroslawa Dabrowskiego Wydzial Inzynierii Ladowej i Geodezji, POLAND

## Abstract

Air pollution caused by suspended particulate matter (PM) remains one of the key environmental challenges in Poland, particularly in the context of public health and spatial planning. This study presents a spatio-temporal analysis based on data from 173 air quality monitoring stations collected between 2015 and 2023. Advanced unsupervised clustering methods based on the Dynamic Time Warping (DTW) metric were applied to identify spatial patterns of pollution at both daily and annual timescales. Based on over 13 million observations, four macroregions were delineated, along with a set of sixteen clusters allowing for assessment of local anomalies. A significant variation in median *PM*_10_ concentrations has been observed across macroregions, ranging from 19.7 to 27.18 $\mu g/m^{3}$. The results revealed the significant role of topographic, urban, and microclimatic factors in shaping the spatial distribution of particulate matter. Urbanized areas in southern Poland (Silesia, Lesser Poland) formed distinctly isolated clusters with high PM levels, in contrast to the stable, low-emission northern lowlands. The analysis further demonstrated that regional policies may be ineffective without supra-regional coordination. These findings show the need of including the high-resolution data analyses into environmental and public health planning to effectively limit the impacts of air pollution.

## Introduction

Air pollution caused by particulate matter (PM) represents a significant challenge, both in terms of public health and in the context of developing future urban environments with particular focus on concepts such as smart cities [1]. Clean air, efficient transportation, access to education, and recreational spaces are the basis of the concept of a smart city [2]. The research proved links between PM exposure and the prevalence of neurodegenerative diseases, particularly Alzheimer’s and Parkinson’s [3]. This concern is especially relevant for countries in the European Union (EU), including Poland, where aging populations are predicted to lead to higher proportions of elderly individuals compared to the younger group [4]. Addressing air pollution is thus critical not only for immediate urban planning and management but also for the long-term planning of healthcare systems and social frameworks within the smart city concept.

Monitoring air quality according to EU regulations usually involves advanced reference sensors, which employ gravimetric methods to measure PM1, *PM*_2.5_, and *PM*_10_ concentrations with high accuracy. These methods are considered the standard for official reporting but are associated with significant installation and maintenance costs [5]. Consequently, their deployment is often spatially limited, restricting the scope of sampling and analysis. In contrast, lower-cost laser-based sensors (LCS) offer a more affordable alternative, but their reduced accuracy renders them unsuitable for regulatory compliance [6].

The EU has established directives 2004/107/EC [7] and 2008/50/EC [8] to define air quality standards and mandate that member states designate zones where air quality is assessed and made publicly accessible. When air pollution levels exceed permissible thresholds, member states are required to implement corrective measures. The European Council emphasizes that *PM*_2.5_ poses the greatest health risk to Europeans due to its ability to penetrate deeply into the respiratory system and bloodstream. Although air pollution levels across Europe have shown a clear downward trend, 97% of the EU population remains exposed to *PM*_2.5_ concentrations that exceed World Health Organization (WHO) guidelines. The European Environment Agency (EEA) identifies energy production, agriculture, manufacturing, road transport, and waste management as the primary sources of *PM*_2.5_, with similar contributions observed for *PM*_10_. Poland faces particular challenges related to PM pollution. Despite national and EU regulatory efforts, the country remains among the most polluted in Europe. Two of the four EU cities that exceeded *PM*_2.5_ limits between 2021 and 2022 are in Poland [9]. Many Polish cities - including Krakow - rank among the world’s most polluted urban areas due to the country’s energy mix based on coal production and geographic conditions that can intensify the retention of pollution [10]. This phenomenon is particularly noticeable during the cold period when the air temperature hovers around 0 degrees Celsius [11].

According to the Central Statistical Office of Poland, the country’s natural population growth rate is –3.9 per 1,000 inhabitants, significantly lower than the EU average of –2.9. This demographic trend suggests potential future strain on healthcare and social systems, particularly given the health impacts of PM exposure and the increasing prevalence of age-related diseases such as Alzheimer’s [12]. Poland’s average life expectancy of 81.1 years for women and 73.4 years for men highlights the urgency of addressing long-term health risks associated with air pollution. In 2022, over 40% of Poland’s greenhouse gas emissions originated from coal-based energy production, while transport and other energy-related sectors each contributed 18%. Poland ranks 21st among 27 EU countries in the share of renewable energy in its energy mix [13].

Research examining urban air quality in Poland indicates that while pollutant concentrations in general have decreased significantly 16 to 34% between 2005 and 2021 - the reductions remain insufficient to meet EU standards, particularly for *PM*_2.5_ and *PM*_10_. Progress has been made since 2011, but overall levels of air pollution remain high (besides ozone). The slow implementation of air quality improvement strategies underscores the need for more decisive actions to align with EU directives and mitigate public health risks [14]. The geographical characteristics of Poland, including its lowlands and industrialized urban regions, play a crucial role in the persistence of high PM concentrations. Combined with its coal-dependent energy system, these conditions create substantial challenges for reducing pollution levels. While stricter industrial regulations and cleaner energy sources are essential, enhanced air quality monitoring networks are also critical for effective spatial and temporal analysis of pollution trends [11].

This study focuses on the spatio-temporal analysis of PM pollution in Poland which is a high-exposure country within the EU. The findings aim to help form strategies for improving urban air quality, supporting public health, and integrating clean environment policies into the broader framework. In this study, data from 173 reference monitoring stations were used. Sensors located throughout Poland were analyzed for the 2015-2023 period to perform a spatio-temporal analysis of *PM*_10_ and *PM*_2.5_ pollution. Although *PM*_10_ data from earlier years are also available, the period from 2015 onward was selected as representative. In 2014, the EU Air Quality Directive (2008/50/EC) was implemented in Poland, standardizing measurement and reporting procedures in accordance with European law. Additionally, studies indicate that 2014 marked a slowdown in the rate of air pollution reduction at the national scale, providing a more stable baseline for assessing the current spatial structure of *PM*_10_ macroregions.

The novelty of this research lies in the first-time application of big data and machine learning techniques to establish air pollution macroregions (APMR) within the country. In this paper, the term air pollution macroregion refers to a geographical subdivision identified through the spatial clustering of areas exhibiting similar air pollution characteristics. A macroregion encompasses an area larger than a single city, county, or even voivodeship, defined by common patterns in air pollutant concentrations that may be related to regional emission sources, prevailing meteorological conditions, and topographic influences. These interrelated factors create a coherent, large-scale spatial unit that reflects the regional dynamics of air quality and atmospheric processes. In contrast, an air pollution microregion represents a smaller, more localized subdivision within a macroregion, characterized by finer-scale variations. A representative example of an air pollution microregion is the Kraków metropolitan area, which displays well-documented, spatially confined pollution patterns influenced by both urban morphology and surrounding terrain [5,10,15]. Specifically, the study included an evaluation of the utility of individual sensor data, which ultimately narrowed the data set to 127 *PM*_10_ and 56 *PM*_2.5_ stations that met the data reliability criteria, forming the basis of a reference database. Advanced imputation of missing values in the time series was performed using hierarchical clustering methods to account for spatial dependencies. Unsupervised machine learning algorithms were employed for regional analysis, leveraging spatio-temporal clustering with dynamic time warping (DTW) to capture both - time and temporal variations. The analyses utilized multiple temporal resolutions of the input data, including daily, and annual averages, to identify patterns across different time scales. Furthermore, multi-year analyses of *PM*_2.5_ / *PM*_10_ ratios were performed, providing information on the sources and composition of particulate matter.

## Materials and methods

### Localization

Poland is a central European country, located on the North European Plain. To the North extends the Baltic Sea, to the South lie Carpathian Mountains and Sudetes. Both act as natural borders from Poland’s southern neighbors. Major part of Poland are lowlands, which take up over 75 percent of the countries surface and are located in central and north regions of the area. The rest of the surface is filled with highlands and mountain ranges in the South. The average altitude is 173 meters above sea level. The total area is 322 575 square kilometers. Poland is located in a temperate warm transitional climate. It is surrounded by different climates, such as marine in the West, continental in the East, cool temperate in the North and Mediterranean in the South. The movement of large air masses, that flow into Poland from all directions has an influence both on climate and levels of *PM*_10_ and *PM*_2.5_ [16]. On the one hand, it cannot be excluded that these air masses inflow significant amounts of air pollutants. On the other hand, they can also outflow them. On a micro-scale: one city and its surrounding terrains. It has been shown that topography of the studied terrain may have major impact on *PM*_10_ and *PM*_2.5_ levels. For example, Krakow is located in a depression surrounded by higher elevation to the North and South. This unfortunate position along with unfavorable meteorological conditions may cause entrapment of air pollutants [15]. This sort of disadvantageous circumstances are likely to occur in many more places all over the country, and may cause extremely high levels of pollution. It’s worth considering these variables on a much larger scale when analyzing levels of air pollution. Topography has major influence in shaping wind patterns by creating natural barriers and corridors which in turn affect inflow and outflow of air masses [17].

Data used in this study was collected from 173 stations in total [18]. 109 of them collected only *PM*_10_ data, 15 gathered only *PM*_2.5_ data and the remaining 49 - both. Even though measurements were conducted since January 2000, the time frame of analyzed data begins in January 2015 and ends December 2023. This certain time frame was chosen due to large proportion of stations not conducting research constantly. For various reasons stations have been halting and starting research in irregular fashion over the years. In latter workflow phases it resulted in having little to none data over large segments of studied area, if longer time periods were chosen.

### Data pipeline

There were certain steps involved with the machine-learning pipeline to provide reliable results based on big data used in this study. Data were collected from 173 stations over 9 years in hourly intervals. In total there were 78840 unique observations with 173 features which gave 13,639,320 cells of data. The big-data machine learning pipeline begins with data collection from GIOŚ (Główny Inspektorat Ochrony Środowiska) archives website [18]. It is publicly available data, shared every year in packages of .xlsx files. Besides *PM*_10_ $\mu g/m^{3}$ and *PM*_2.5_ $\mu g/m^{3}$, other measurements can be found, like carbon monoxide *mg*/*m*^3^, benzene *ng*/*m*^3^, or sulfur dioxide $\mu g/m^{3}$. There are two types of measurement frequencies shared with the public: every 24 hours and 1 hour. Through data transformation, different intervals can be calculated using upscaling or downscaling methods. Final results were calculated at yearly and daily intervals.

**Fig 1:**
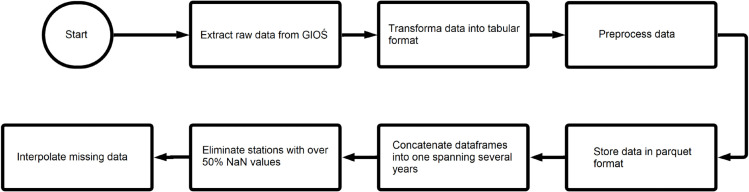
Data preparation pipeline.

An observation is a measurement of a specific type of air pollution taken by a given station at a particular moment. A few challenges were recognized during prepossessing that were related to data ingestion from the origin source and their non-uniqueness in names. Also before loading to the final database data cleaning was applied to make sure that all observations were in numeric formats. The cleaning process focused on aligning the data into the correct tabular format with consistent naming across all sources (see Fig 1).

NaN values represent data that were unavailable at a given time and location. Stations with more than 50% of NaN values were excluded from the analysis (see Fig 1). To clarify the rationale behind selecting the 50% utoff threshold, consider two alternative scenarios:

1. **A 10% cutoff** - meaning that only stations with up to 10% of NaN values (at least 90% of valid data) would be retained. This approach would ensure very high data quality; however, it would significantly reduce the number of available stations, potentially increasing spatial bias and lowering representativeness.
2. **A 90% cutoff** - meaning that stations with up to 90% of NaN values (as little as 10% of valid data) would still be included. While this would retain a much larger number of stations, many would contain excessive missing data, introducing noise and likely distorting the final results.

In conclusion, the 50% threshold represents a balanced compromise between retaining a sufficient number of stations and maintaining acceptable data quality.

**Fig 2:**
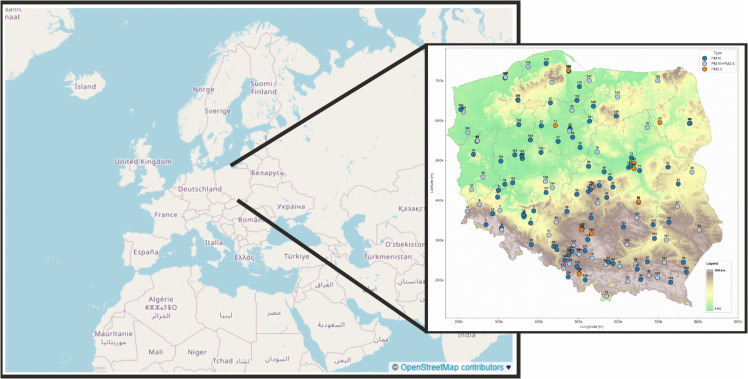
Geographic distribution of air pollution monitoring stations included in the study (January 2015 – December 2023) – *PM*_10_ (dark blue), *PM*_2.5_ (orange), $PM_{10}\;+\;PM_{2.5}$ (light blue). The background map is based on OpenStreetMap data [19]. (hypsometric map from WMTS: [20]; Ref. System: EPSG 2180).

**Fig 3:**
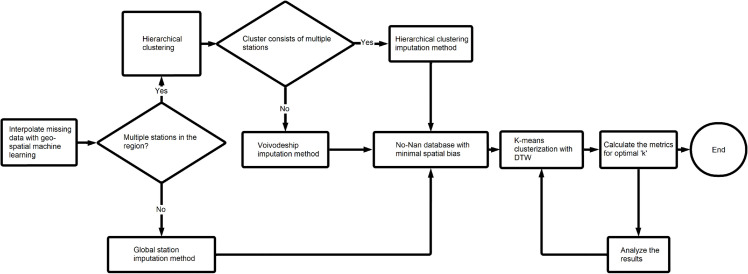
Data imputation and clustering pipeline.

The remaining 140 stations (see Fig 2) still contained significant number of NaN values. This problem was solved using the hierarchical clustering imputation method Fig 3. This approach was significantly better than conventional methods, such as interpolation based on a single station. The spatiotemporal nature of the data requires a solution that minimizes potential spatial bias - for example - air pollution profiles may differ between the southern and northern parts of the country.

Began with calculating Pearson’s [21] correlation matrix for each voivodeship individually, which helped identify local similarities between stations 1.

$$r=\frac{\sum_{i=1}^{n}(x_{i}-\bar{x})(y_{i}-\bar{y})}{\sqrt{\sum_{i=1}^{n}(x_{i}-\bar{x}{)}^{2}\sum_{i=1}^{n}(y_{i}-\bar{y}{)}^{2}}}$$ (1)

where *n* is the number of observations, *x*_*i*_ and *y*_*i*_ represent the values of the respective variables, and $\bar{x}$ and $\bar{y}$ are their arithmetic means.

Then, hierarchical clustering using Ward’s method [22] was applied to the correlation matrix of each voivodeship to identify groups of similar time series within the region.

**Fig 4:**
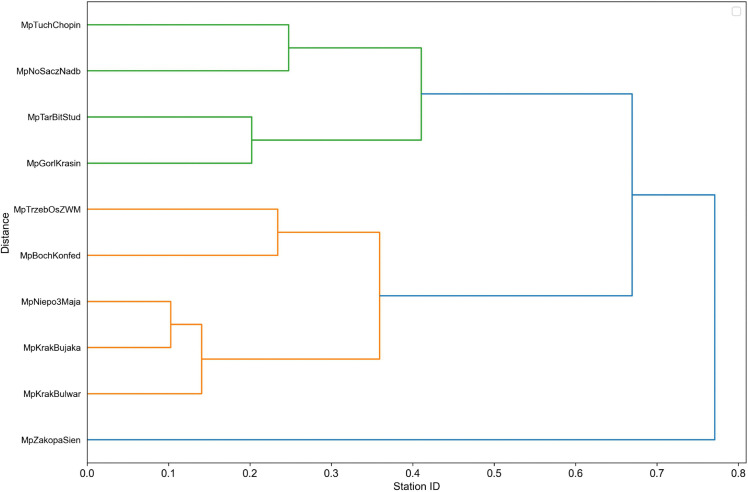
Dendogram of clusters from lesser Poland Voivodeship.

This method is an unsupervised machine learning technique, utilizing an agglomerative approach. Initially, each time series is treated as a cluster. Next, the algorithm searches for the two most similar clusters and merges them into one. This step is repeated until all data points are in one big cluster. Finally, the dendrogram (Fig 4) is created. It is a visual representation of the clustering process. Upon review, we can conclude the level of similarity of each cluster and how they were connected in each iteration. Even though a dendrogram was available for each voivodeship, only one was reviewed manually.

The decision was made to set the cutoff distance at half of the max tree height automatically for every region. This solution gave optimal results in a relatively short amount of time, however, better results could have been achieved through exploratory data analysis. Once the algorithm chose groups in a given region, the within-cluster average value was calculated for each row and used for imputation. However, this implies having more than one station in each cluster, which wasn’t always the case. Two edge cases, have to be considered. The cluster consists of one time series, but there are other stations in the region. Second, there aren’t any other stations in the region. The first case is handled by calculating the within-region mean value by row and imputing that for standalone clusters. Second is imputed with the mean value of the whole column for a given station. This method of filling in the gaps, introduced spatial dimension into the analysis, as the data was imputed only geo-locally. The potential spatial bias which could negatively affect the final results, has been minimized. For example, the measurements in the North may differ in nature from stations in the South. Therefore, it would be unwise to use data from one to impute in the other. The main difference between hierarchical clustering and k-means clustering is choosing the number of clusters. K-means [23] has a priori and hierarchical a posteriori approach, essentially one needs the number of clusters before executing, other doesn’t.

### Unsupervised machine learning

Machine learning has seen significant gain in recognition in the last few years, due to the rise in popularity of artificial intelligence [24]. Along with the rapid increase in data availability and quantity in general [25]. Machine learning became essential for any sensible data analysis and modeling, both in scientific [26] and commercial [27] workflows. Machine learning serves various purposes in data science [28]. It is a helpful tool in every part of the data pipeline, ranging from data cleaning and imputation to final business intelligence results [29].

Machine learning can be divided into unsupervised and supervised. Supervised is most commonly used for classification and regression problems. It needs labeled data to train the model. Labeling often has to be done by humans. Thus obtaining data for supervised learning can be very expensive and time-consuming. It uses different metrics for model evaluation, due to the nature of the data it works on. This enables assessment of the models accuracy, and how well it explains a given feature. In turn, unsupervised learning trains on unlabeled data and its primary task is to find unknown patterns within the data. These patterns are often imperceptible to the human eye, often due to vast amounts of data. Thanks to this it can be a very valuable input in scientific research. One of its main strengths is minimal human intervention. The algorithm only needs clean and representative data. It works out the hidden structure of the data based on the similarities and dissimilarities within and yields the desired results. The outcome then can be verified by researchers, which are given the choice to either accept the result or adjust the data and parameters. The biggest drawback of unsupervised machine learning is that it is virtually impossible to decide whether the returned results are correct. Since there is no labeled data, there is nothing to constitute correctness, as in the case of supervised learning.

K-means algorithm is an unsupervised learning algorithm. It categorizes data points into clusters based on the distance between data points and a cluster center. The most commonly used distance metric is the Euclidean distance. However, due to the nature of the data in this study which is time series data, we employed Dynamic Time Warping (DTW). From a performance perspective, the Euclidean distance is computationally efficient, with linear time complexity 𝒪(n), but it lacks robustness to temporal misalignments and variations in sequence length. In contrast, DTW provides higher accuracy in matching time series that exhibit phase shifts or non-linear temporal variations by allowing flexible alignments, though at the cost of increased computational complexity $𝒪(n^{2})$ and higher memory consumption. K-means is a well-established machine learning algorithm. It has many advantages over different methods. It’s very fast, eq. it’s faster than the previously mentioned hierarchical clustering, especially in its simplest form (using Euclidean distance). Adaptability, there are many variations of the original algorithm, such as fuzzy c-means [30] or k-means++ [31].

Simplicity, the algorithm can be broken down into 5 steps.

1. Select the number of clusters ‘k’ to identify within the data. That is k in k-means.
2. ‘Randomly’ initialize centroids.
3. Assign each data point to the closest cluster. Assignment of new cluster is given as Eq (2) solely for Euclidean Distance.
  $$c_{i}=\arg\min_{j=1}^{k}||x_{i}-\mu_{j}|{|}^{2},$$ (2)
  *c*_*i*_ is the newly assigned cluster to the point *x*_*i*_. $\mu_{j}$ is the centroid-point. $||x_{i}\;-\;\mu_{j}|{|}^{2}$ is the euclidean distance between the point and centroid. *k* is the a priori set number of clusters from step 1
4. Calculate the mean value of each cluster and set that as a new centroid. This is expressed by Eq (3)
  $$u_{j}=\frac{1}{n_{j}}\sum_{i=1}^{n}x_{i}[c_{i}=j],$$ (3)
  *u*_*j*_ is centroids new location, $\frac{1}{n_{j}}\sum_{i=1}^{n}x_{i}[c_{i}=j]$ is the mean value of the *j* cluster, because *n*_*j*_ is the count of points in *j* cluster and $\sum_{i=1}^{n}x_{i}[c_{i}=j]$ is the sum of the point-values inside the *j* cluster.
5. Repeat steps 3 and 4 until the clusters stop changing or max-iteration value is reached.

It should be emphasized that the equations above are true if k-means uses Euclidean distance as the similarity measure. Although, in general, it is the most common metric. In this study, the Dynamic Time Warping metric (DTW) was used. According to [32] DTW can find optimal global alignment between the time series. It can capture the similarity between two temporal sequences varying in speed or with time shifts, unlike Euclidean distance. Thus it is much better at pattern recognition in the temporal sequences domain. It’s especially important in studies like this, to recognize trends and global patterns over long periods. DTW, as the name suggests ‘warps’ time sequences in a way to locally minimize pairwise distance between two moments in time. Given two time series, lets compare it with the simpler similarity measure.

$$X=\{x_{1},x_{2},\ldots ,x_{n}\}$$ (4)

$$Y=\{y_{1},y_{2},\ldots ,y_{n}\}$$ (5)

The Euclidean Distance between them would be expressed with Eq (6)

$$d(X,Y)=\sqrt{\sum_{i=1}^{n}(x_{i}-y_{i}{)}^{2}}$$ (6)

Where $\sqrt{\sum_{i=1}^{n}(x_{i}-y_{i}{)}^{2}}$ is the square root of sum of squared differences for each *i* pair of the sequence.

DTW is a bit more complicated, because of the optimization process that synchronizes two originally un-synchronized sequences. In simple terms, the algorithm seeks out best possible match for each point in the two time series in order do minimize the distance between them. The distance measure does not have to be euclidean, any can be used.

The DTW distance between two time series *X* and *Y* can be expressed by Eq (7)

$$d_{DTW}(X,Y)=min_{\alpha ,\beta }\sum_{i=1}^{n}||x_{i}-y_{\alpha (i)}||+||y_{i}-x_{\beta (i)}||,$$ (7)

where α and β are warping functions that specify how the time indexes of the series should be transformed, and *n* is the length of the series.

Using the formula above, the K-means algorithm can be modified by replacing the Euclidean distance with the DTW distance. The process of assigning points to the clusters using DTW is given as Eq (8)

$$c_{i}=\arg\min_{j=1}^{k}d_{DTW}(x_{i},\mu_{j})$$ (8)

### Clustering evaluation metrics

The first and arguably the most important step involved with k-means clustering is choosing the ‘k’ number of clusters. Sometimes, a researcher can decide on the number of distinct patterns within the data, which would be equivalent with the number ‘k’. However, when dealing with large scale datasets it is almost impossible to decide just based on exploratory data analysis done by hand. That is why, many useful evaluation metrics have been developed to help recognize the ‘right’ number of clusters based on traits like within cluster similarity or total dispersion. In this study, four metrics were used: the elbow method, Calinski-Harabasz index, Davies-Bouldin index and Silhouette coefficient. Although time-series clustering was applied, the primary objective was to identify spatial and temporal dependencies rather than to analyze temporal similarity per se. For this reason, DTW was employed to account for temporal misalignments and amplitude variations within daily PM profiles, while classical clustering validity indices such as the Davies–Bouldin (DB), Calinski–Harabasz (CH), and Within-Cluster Inertia were subsequently used to evaluate the compactness and separability of the resulting groups. This approach allowed the integration of time-series sensitivity with interpretable, geometry-based evaluation metrics, ensuring both temporal flexibility and spatial coherence of the macroregional clusters. In cluster analysis, the formation of clusters is guided not only by quantitative metrics but also by the domain knowledge of the interpreter. Selecting a single, “optimal” number of clusters in an entirely objective manner is extremely challenging. Therefore, in this study, multiple metrics were employed, which may occasionally yield conflicting results. However, when combined with expert knowledge, the analysis of clustering results on maps and their subsequent interpretation can serve as a reliable and meaningful identifier [33].

#### Elbow method.

The elbow method [34] is a method to recognize significant turning point in sum squared error (SSE) between centroid and points inside the cluster. The SSE is plotted against the number of clusters. Goal is to choose *k*, that sees a sudden drop of SSE and then plateaus.

$$SSE=\sum_{i=1}^{k}\sum_{x\in C_{i}}||x-\mu_{i}|{|}^{2},$$ (9)

where *k* is the number of clusters, *C*_*i*_ is the *i*th cluster, $\mu_{i}$ is the centroid of the *i*th cluster, and $||x\;-\;\mu_{i}|{|}^{2}$ is the squared distance between point *x* and the centroid $\mu_{i}$.

#### Calinski-Harabasz index.

Calinski-Harabasz index (CH) [35] is a ratio of between cluster separation and individual cluster cohesiveness. Basically, it’s a index of how well separated the clusters are and how ‘dense’ they are. It is calculated by the equation below (10):

$$CH=\frac{t(B_{k})}{t(WI_{k})}\times \frac{n_{W}-k}{k-1},$$ (10)

where *t*(*B*_*k*_) it the trace of the covariance matrix between clusters, *t*(*WI*_*k*_) it the trace of the covariance matrix within the cluster, *n*_*W*_ is the total number of the data points, *k* is the number of clusters.

#### Silhouette index.

The Silhouette Coefficient (SC) evaluates how well data points fit their assigned clusters by considering both their internal cohesion within the cluster and their separation from other clusters. It ranges from –1 to 1, where –1 indicates that the point would fit better in a different cluster, 0 suggests that the point lies on or near the boundary between two clusters, and 1 signifies a perfect fit to the assigned cluster.

$$SC=\frac{b-a}{max(a,b)},$$ (11)

where *a* is the mean distance between the point and other points inside the cluster and *b* is the distance between a point and the nearest cluster (to which this point does not belong).

#### Davies Bouldin index.

The Davies-Bouldin Index (DB) evaluates the internal compactness of clusters and their separation from one another. The DB ranges from 0 to infinity, where lower values indicate better clustering quality [36]. The DB is defined by the equation below (12).

$$DB=\frac{1}{k}\sum_{i=1}^{k}\max_{i\neq j}\frac{s_{i}+s_{j}}{d_{ij}},\;\;\;i=1,\ldots \ldots ,k,$$ (12)

where *s*_*i*_ is the average distance between the *i*-cluster centroid and points in this cluster, *s*_*j*_ is the average distance between the *j*-cluster centroid and points in this cluster and *d*_*ij*_ is the distance between *i* and *j* cluster centroids.

## Results

### Optimal cluster choice

The optimal cluster choice was based on the previously mentioned four metrics. The focus was primarily on local extremes. For the sake of improved interpretability the metrics were scaled using the min-max method. Moreover the optimal ‘k’ has to be an intersection of metrics concerning all three types of results: *PM*_10_ *PM*_2.5_ and *PM*_2.5_ to *PM*_10_ ratio. The metrics were calculated on the daily interval data. The possible k’s were within the range of 2 to 20. A key assumption is that k in the range of 2 to 5 was not considered during the analysis of the metrics due to the attempt to identify macroregions.

**Fig 5:**
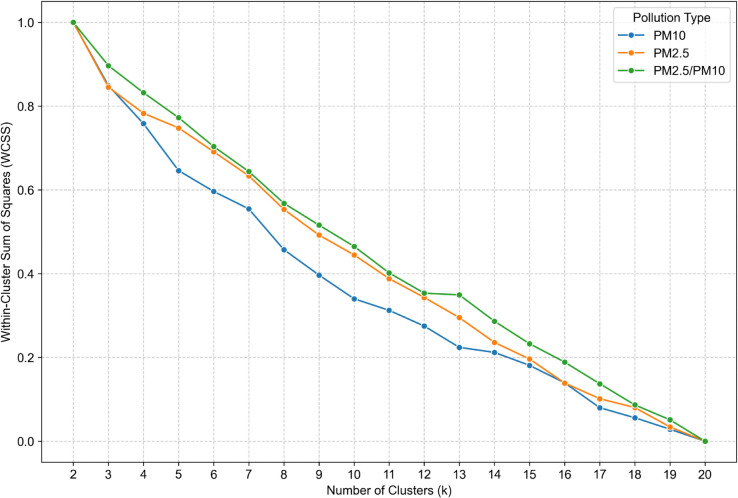
Within cluster sum of squares metric for cluster number validation for *PM*_10_ (blue line), *PM*_2.5_ (orange line), *PM*_2.5_/*PM*_10_ (green line).

The analysis of within cluster sum of squares against the number of clusters involves recognizing a significant turning point called the elbow. Results showed in (Fig 5) are in a steady decline, thus it is impossible to find an elbow, especially across all three types. This metric won’t be helpful for this case.

**Fig 6:**
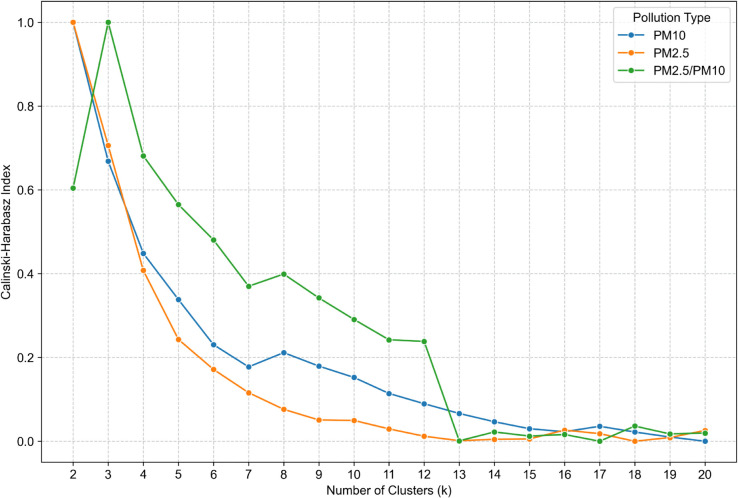
Calinski-Harabasz Index for cluster number validation for *PM*_10_ (blue line), *PM*_2.5_ (orange line), *PM*_2.5_/*PM*_10_ (green line).

Calinski-Harabasz index (Fig 6) provides more valuable information. The k equal to 8 is a local maximum, that indicates better clustering for both *PM*_10_ and PM ratio. Also *k* equal to 12 is interesting because of the sudden decline in PM ratio index value. The index stabilizes for *k* in the range of 13 to 20, where slight fluctuations occur. In general the Calinski-Harabasz index is almost in a monotonic decline, which suggests worse clustering with the k value increase.

**Fig 7:**
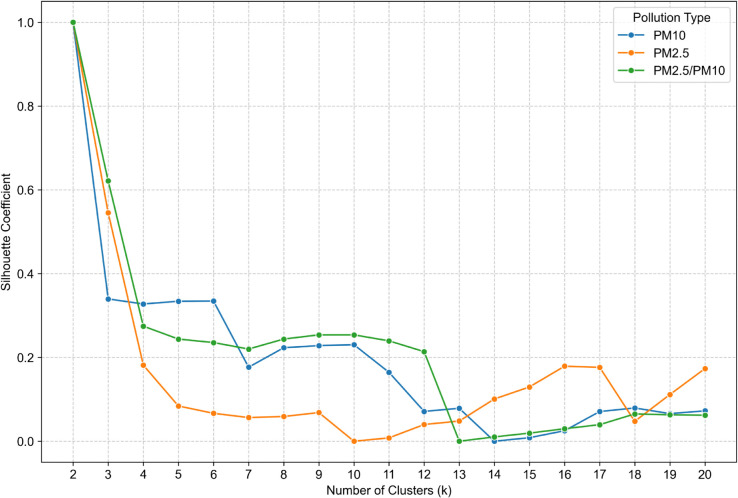
Silhouette for cluster number validation for *PM*_10_ (blue line), *PM*_2.5_ (orange line), *PM*_2.5_/*PM*_10_ (green line).

The silhouette (Fig 7) exhibits significant variance with a fairly consistent pattern across the three measurements in the range of k 2 to 12. Similarily to Calinski-Harabasz index, there is a significant drop of PM ratio between k 12 and 13. There is a local minimum on k 7 and a local maximum on k 9 for all three types. In the range of k 13 to 20, there are no consistent local extrema. One noteworthy is a k 16 local maximum for PM 2.5.

**Fig 8:**
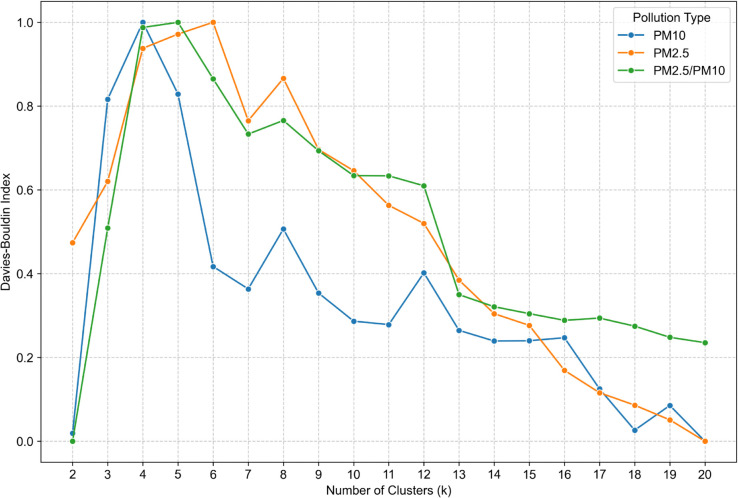
Davies-Bouldin Index for cluster number validation for *PM*_10_ (blue line), *PM*_2.5_ (orange line), *PM*_2.5_/*PM*_10_ (green line).

When analyzing the Davies-Bouldin index (Fig 8), the focus was on identifying local minima, which indicate better clusterings. The k 7 was one, which was highly distinct. It is a good clustering indicator for all types of measurements. On the other hand k 8, indicates bad clustering which goes against the Calinski-Harabasz index.

A comprehensive evaluation of all clustering metrics led to the selection of k = 7 as the most well-justified cluster size. This choice was predominantly influenced by the Davies-Bouldin index, which strongly indicated a high-quality partitioning of the data. The second configuration, k = 16 was considered based on the assumption that the resulting cluster structure would closely correspond to the distribution of voivodeships. The choice of 16 clusters was guided by external knowledge and expert judgment, aiming to balance granularity with interpretability.

### The cluster maps

To identify the macroregions of air pollution in Poland, clustering results were visualized across 12 maps. These include two sets of maps, each based on daily and annual data, corresponding to the previously selected cluster sizes of *k* = 7 and *k* = 16.

**Fig 9:**
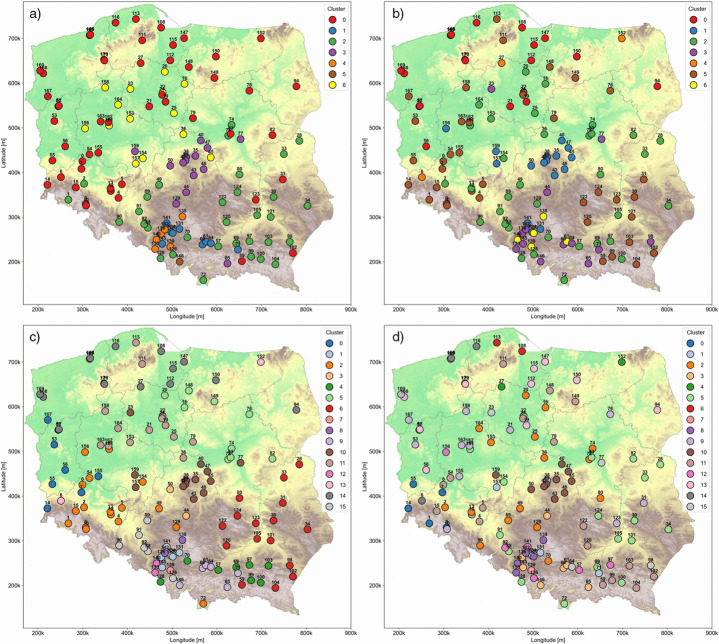
*PM*_10_ station cluster distribution (hypsometric map from WMTS: [20]; Ref. System: EPSG 2180). a) *PM*_10_, daily, k = 7. b) *PM*_10_, yearly, k = 7. c) *PM*_10_, daily, k = 16. d) *PM*_10_, yearly, k = 16.

The cluster distribution (Fig 9a) (*PM*_10_, daily, k = 7) is dominated by two major clusters: 0 and 2. Cluster 0 covers most of northern and western Poland, where the terrain is predominantly lowland. Cluster 2 spans the southeastern part of the country and partially overlaps with clusters in Silesia and Małopolska, where the terrain is mostly highland. Overall, the clusters divide Poland along a northeast–southwest axis. Clusters 3 and 6 are located in central Poland but differ geographically: Cluster 3 corresponds mainly to the transition zone between the Przedborska and South Masurian Highlands and the central lowlands, while Cluster 6 is associated with river valleys (e.g., the Noteć Valley) and the lake district (Pojezierze region). Clusters 1, 4, and 5 are concentrated in Silesia and Małopolska, primarily around major urban areas. In summary, daily *PM*_10_ clusters exhibit distinct spatial patterns and clear separation between regions.

The cluster distribution shown in (Fig 9b) (*PM*_10_, yearly, k = 7) appears more chaotic than its daily counterpart. Most of the country is dominated by clusters 0, 2, and 5, which are highly interwoven and encircle the remaining smaller clusters. Cluster 0 is primarily associated with lowland areas and the lake districts in the north. In contrast, clusters 2 and 5 do not correspond to specific geographical regions, suggesting that their grouping may be influenced by factors other than topography. Cluster 1 forms a compact group centered around the Łód ź Voivodeship, particularly in the Wyżyna Przedborska region. It also includes six additional stations located to the south and west, most of which are situated in upland areas. Clusters 3 and 6 are concentrated around major urban centers such as Kraków and Katowice—regions typically characterized by elevated air pollution levels, especially during the colder months. Cluster 4 consists of three stations, all sharing a common feature: close proximity to bodies of water.

Notable similarities in cluster distribution can be observed between (Fig 9c (*PM*_10_, daily, k = 16) and (9b). In particular, clusters 13 and 10 in (Fig 9c) closely resembles clusters 4 and 1 in (Fig 9b), respectively. This resemblance suggests not only analogous local variations but also shared long-term trends among the corresponding stations. Overall, the cluster distribution in (Fig 9c) is relatively compact, with each cluster largely confined to a specific geographical region. Clusters 6 and 4 are located in highland areas near the southeastern border. Cluster 15 is situated along a river basin of Odra river, with the exception of station 126, which deviates from this pattern. Clusters 14, 5, and 11 are composed of stations situated in the northern and center lowlands.

The long-term trends captured in the annual *PM*_10_ data do not result in many spatially coherent clusters. The distribution presented in (Fig 9d) (*PM*_10_ yearly, k = 16) appears disorganized, making it difficult to analyze or identify consistent characteristics within individual clusters. Notable exceptions are clusters 4 and 10, which show similarities to clusters identified in the previous maps. Most clusters in this dataset are either small, comprising only 2 to 4 stations and/or are dispersed widely across the country, further complicating spatial interpretation.

**Fig 10:**
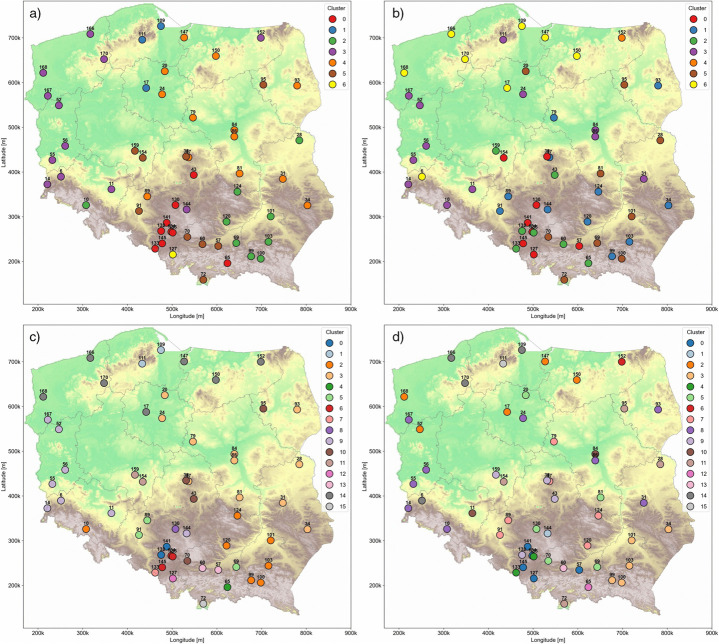
*PM*_2.5_ station cluster distribution (hypsometric map from WMTS: [20]; Ref. System: EPSG 2180). a) *PM*_2.5_, daily, k = 7. b) *PM*_2.5_, yearly, k = 7. c) *PM*_2.5_, daily, k = 16. d) *PM*_2.5_, yearly, k = 16.

Cluster 4 in (Fig 10a) (*PM*_2.5_, daily, k = 7) largely follows the course of major river basins-primarily the Wisła—with the exception of stations 93, 89 and 150, which deviate from this pattern. Cluster 6 consists of a single station (127), potentially indicating unique *PM*_2.5_ pollution levels at that location. Cluster 2 is associated with elevated terrain in the south, with most of its stations situated in the Podgórze Środkowobeskidzkie and Kotlina Sandomierska. Cluster 5 includes stations distributed across all major geographical regions, however, these stations are generally located near terrain depressions or low-lying areas. Cluster 0 is concentrated on the Wyżyna Ślaska, with two outlier—stations 43 and 65—also located in upland regions. Clusters 1 and 3 dominate the northwestern part of the country, an area characterized by lowlands interspersed with forests and numerous lakes.

Cluster 0 exhibits in (Fig 10b) (*PM*_2.5_, yearly, k = 7) a distribution similar to its counterpart in (Fig 10a), though it now slightly overlaps with cluster 0 in the Wyżyna Ślaska region. Cluster 1 is distributed across upland and basin areas in the southeastern part of the country. Clusters 0, 1, 2, and 5 are primarily located in the southern highlands, gradually extending into the central lowlands and to a lesser extent, overlapping with northern regions. Compared to (Fig 10a), the clusters in the south are less compact and more fragmented. In contrast, cluster 6 forms an almost perfectly delineated region, with the exception of station 6, which appears to be an outlier. This cluster comprises of stations situated near renewable energy sources, such as wind and solar power plants. Cluster 3 is predominantly distributed across the central lowlands in a vertical pattern. Due to its large spatial extent, identifying a single unifying characteristic for this cluster is challenging. Finally, cluster 4 is a standalone group consisting of a single station—station 152—which, as previously noted, tends to form small, isolated clusters.

The cluster distribution presented in (Fig 10c) (*PM*_2.5_, daily, k = 16) reveals several notable features and exhibits clear spatial separation across the country. Most clusters are geographically compact, with the exception of clusters 10, 2, and 5, which are more dispersed. It is particularly noteworthy that cluster 2 closely mirrors its distribution from Fig (10a), suggesting stable spatial characteristics reflected in local temporal changes. Cluster 3 shows strong alignment with the course of the Wisła River, although station 93 stands out as an outlier. In the southern highlands, numerous clusters have formed in close proximity. Clusters 0 and 6 are situated near the Górnoślasko-Zagłebiowska Metropolia, while clusters 13, 4, and 15 are entirely contained within the Małopolska region. As observed in previous maps, the northwestern parts of the country continue to exhibit well-defined and spatially distinct clusters.

(Fig 10d) (*PM*_2.5_, yearly, k = 16) illustrates that spatial separation is primarily influenced by similarities in local temporal variations among stations. The overall distribution is visibly chaotic, with many clusters overlapping, often within the boundaries of just one or two geographical regions. Clusters 2, 5, 8, 11, and 15 exemplify this lack of a clear spatial pattern, highlighting the irregularity in cluster formation. Once again, station 152 forms a standalone cluster, consistent with its behavior observed in (Fig 10b).

**Fig 11:**
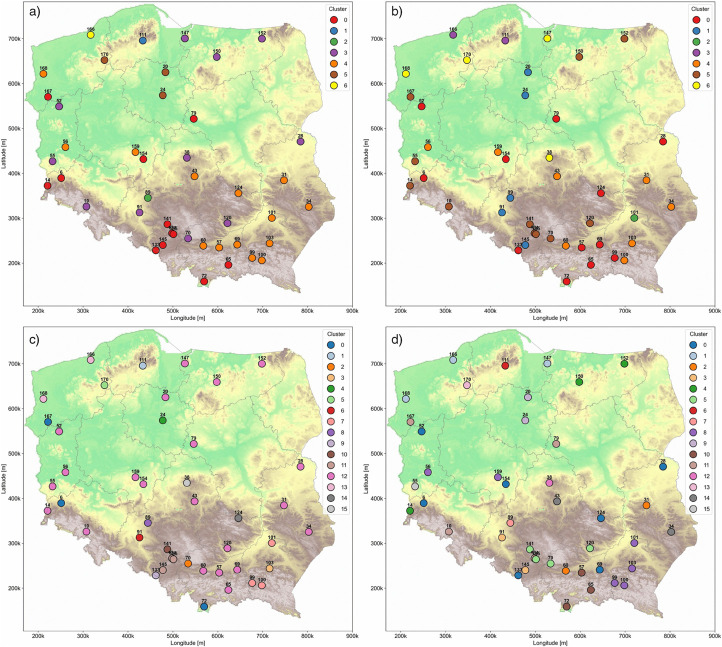
*PM*_2.5_/*PM*_10_ station cluster distribution (hypsometric map from WMTS: [20]; Ref. System: EPSG 2180). a) *PM*_2.5_/*PM*_10_, daily, k = 7. b) *PM*_2.5_/*PM*_10_, yearly, k = 7. c) *PM*_2.5_/*PM*_10_, daily, k = 16. d) *PM*_2.5_/*PM*_10_, yearly, k = 16.

The cluster distribution presented in (Fig 11a) (*PM*_2.5_/*PM*_10_, daily, k = 7) is notably distinct from those in the other maps. It features a dominant, large cluster that spans the entire country and serves as a background or baseline layer. In contrast, the remaining clusters are much smaller, each comprising between one and four stations. Cluster 4 exhibits some similarity to Cluster 2 in (Fig 10a and 10c). Cluster 6 consists solely of station 152, which frequently appears as a standalone or part of a small cluster across different maps. Clusters 1, 3, and 5 are each composed of individual stations, located primarily in the central and northern regions of the country.

The clusters shown in (Fig 11b) (*PM*_2.5_/*PM*_10_, yearly, k = 7) exhibit an unusual spatial pattern. Rather than being concentrated in compact regions, they extend over large areas in curved formations, with several noticeable outliers per cluster. Cluster 3 forms a linear pattern that delineates the Pojezierza region in the northwestern part of Poland. Cluster 2 outlines the central lowlands, effectively encircling this geographical area. Cluster 0 is primarily located near the southern border with the exception of stations 52 and 159, which deviate from this pattern. Clusters 1 and 5 appear more randomly distributed, emerging across various geographical zones without a clear or consistent spatial structure.

The cluster distribution in (Fig 11c) (*PM*_2.5_/*PM*_10_, daily, k = 16) follows a pattern similar to that observed in (Fig 11a), albeit on a smaller scale. The dominant cluster is reduced in size but still functions as a background group, encompassing stations that lack a clear geographical association. The remaining clusters are composed of only one to three stations each, making it challenging to identify any distinct spatial characteristics or regional coherence within them.

**Fig 12:**
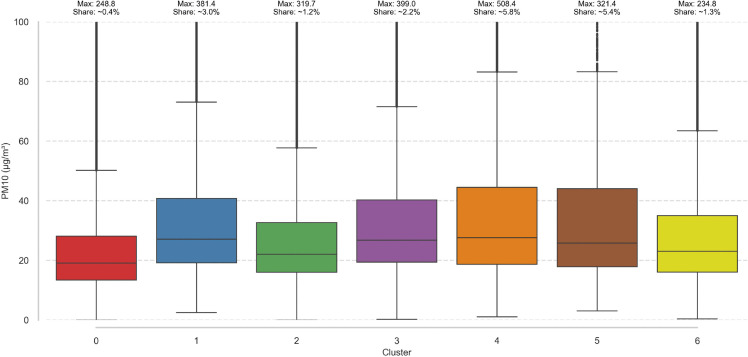
*PM*_10_ daily, k = 7 data distribution within clusters.

The cluster distribution in (Fig 11d) (*PM*_2.5_/*PM*_10_, yearly, k = 16) appears highly irregular. Clusters are either very small, comprising one to three stations, or extend across broad areas of the country. Even the small clusters are widely dispersed, with examples such as clusters 4, 11, and 2 located far apart from one another. Based solely on the terrain and known geographical regions, it is difficult to discern any consistent spatial patterns or logic in the clustering.

(Fig 12) presents the distribution of *PM*_10_ values across clusters identified in (Fig 9a) (*PM*_10_ daily, k = 7). The y-axis is truncated at 100 $\mu g/m^{3}$, with values exceeding this threshold considered outliers. For each cluster, the maximum value is indicated above the corresponding boxplot, along with the percentage of observations classified as outliers. Cluster 0 (northern lowlands) exhibits a notably lower concentration distribution, with a median below 20 $\mu g/m^{3}$ and the fewest outliers among all clusters. Similarly, Cluster 6 (central lowlands) shows a low median and the lowest maximum outlier value. In contrast, Clusters 1, 3, 4, and 5 (southern highlands) demonstrate significantly higher *PM*_10_ concentrations, particularly between the median and third quartile. These clusters also contain extremely high maximum outlier values ranging from 321.4 to 508.4 $\mu g/m^{3}$, with outlier shares reaching up to 5.8%. Cluster 2 (southeastern terrains) represents an intermediate case, with a distribution situated between the low and high concentration groups.

## Discussion

The selection of the optimal number of clusters (k) was informed by a comparative assessment of four established validation metrics: WCSS, Calinski-Harabasz Index, Silhouette Coefficient, and Davies-Bouldin Index. To enhance interpretability and comparability across *PM*_10_ *PM*_2.5_, and *PM*_2.5_/*PM*_10_ ratio datasets, all metrics were normalized using the min-max scaling method. The analysis was conducted on daily-resolution data within the range of k = 6 to 20.

WCSS exhibited a monotonic decline, lacking a clear elbow point, and was thus uninformative. The Calinski-Harabasz Index peaked at k = 8 but declined thereafter, stabilizing beyond k = 13. The Silhouette Coefficient showed consistent structure up to k = 12, with a maximum at k = 9 and a local minimum at k = 7. The Davies-Bouldin Index indicated k = 7 as the most favorable, reflecting optimal cluster compactness and separation. Given the divergence between metrics, a multi-criteria approach was adopted, with k = 7 emerging as the most robust solution. Additionally, k = 16 was selected to explore potential alignment between pollution patterns and Poland’s administrative divisions (voivodeships), aiming to assess whether regional governance, policies, or awareness campaigns may manifest in spatially distinct pollution clusters.

The daily *PM*_10_ cluster map revealed strong spatial structuring, dominated by two major clusters: one spanning northern and western Poland, and the other encompassing the southeast, including Silesia and Małopolska. These clusters align along a northeast–southwest axis. Central Poland was divided between clusters associated with topographic features such as upland transition zones and river valleys. Urban-industrial centers in Silesia and Małopolska formed distinct, well-separated clusters, highlighting the influence of terrain and anthropogenic factors on daily *PM*_10_ levels.

This observation underscores the importance of topography and urban-industrial density in shaping pollution patterns at daily resolution. In contrast, annual mean *PM*_10_ data exhibit less pronounced spatial differentiation. For instance, in Małopolska, stations 60 (Kraków–Bujaka), 61 (Kraków–Bulwarowa), and 64 (Niepołomice), all geographically proximate, belonged to the same cluster in the daily data but diverged significantly in the annual data. Notably, station 61 became part of a geographically isolated southern cluster (cluster 6). The divergence arises directly from the differences in time-series comparisons between annual and daily data. The daily data capture short-term dynamic patterns that account for a substantial portion of the similarities between time series. When the data are aggregated to an annual resolution, these short-term variations are lost, and the focus shifts primarily to long-term trends, disregarding local temporal peaks and shifts. Consequently, this leads to divergence in the clustering results.

A similar divergence is observed in the Silesian Voivodeship, where stations 142 (Zabrze), 136 (Knurów), 140 (Rybnik), and 133 (Godów) form a coherent group in the daily data but separate in the annual data, with station 140 isolated into cluster 6. Interestingly, both stations 61 and 140 are located near small water bodies. Likewise, stations 138 (Myszków) and 139 (Pszczyna) exhibit similar patterns.

These findings suggest that local water bodies may influence long-term *PM*_10_ measurements, potentially due to microclimatic effects or the presence of water vapor acting as a precursor in secondary particle formation. This observation is in line with Tyrso study [37]. However, the impact of water bodies on PM concentrations is not uniform and depends strongly on their spatial relation to urban structures. Evidence from Wuhan suggests a generally positive influence of large water bodies, driven by their scale, absence of emission sources, and restrictions on dense construction [38]. Other research reports both positive and negative effects of rivers depending on environmental conditions [39]. Specifically, proximity to rivers may reduce *PM*_2.5_ through humidity-driven particle deposition once a critical threshold is exceeded. Still, when humidity remains lower, particles are less likely to settle and may persist in suspension. Moreover, under moderately humid conditions, gaseous precursors can enhance secondary particle formation. Thus, the role of rivers in urban air quality is context-dependent, shaped by both morphology and meteorology.

### Macroregional patterns of daily *PM*_10_ clusters in Poland

Daily-resolution data were selected for macroregional classification of *PM*_10_ as annual averages tend to suppress short-term variability associated with anthropogenic activities. In contrast, daily data preserve finer-scale temporal fluctuations, allowing for a more nuanced identification of pollution regimes shaped by both human and environmental drivers. The spatial distribution of daily *PM*_10_ clusters across Poland, as illustrated in Fig 9a, reveals distinct macroregional structures aligned with topographic, climatic, and urban-industrial characteristics. Based on the dominant cluster presence and geographical coherence, five macroregions can be delineated:

1. **Western-Baltic Lowland (Majority of Cluster 0)**: This region encompasses the majority of northern and western Poland, including the voivodeships of West Pomerania, Lubusz, Greater Poland, and parts of Kuyavia-Pomerania, Podlaskie and parts of Warmian-Masurian voivodeship. It is characterized by low population density, relatively flat terrain, and limited industrial activity. The dominance of Cluster 0 indicates generally lower *PM*_10_ levels, likely influenced by favorable dispersion conditions and a lack of large urban emission sources. Cluster 0 is characterized by the lowest average concentration values, with medians below 20 μg/m^3^. In the boxplot, it displays the narrowest interquartile range and only 0.4% of outliers, indicating a relatively minor influence of sudden external factors. This cluster is located in regions with the highest forest cover in the country—Lubusz Voivodeship with nearly 50% forested area, as well as Pomeranian and West Pomeranian Voivodeships, each with approximately 35% [40].
2. **Carpathian Foothill-Upland (Cluster 2)**: Spanning the Carpathian foothills and uplands of Lesser Poland and Subcarpathia, this macroregion exhibits higher *PM*_10_ concentrations associated with Cluster 2. The distribution aligns with complex topography, frequent thermal inversions, and dense urbanization in valleys, contributing to pollutant accumulation. Cluster 2 is characterized by a relatively narrow boxplot, with the upper quartile around 35 $\mu {\text{g/m}}^{3}$ and the lower quartile at approximately 17 $\mu {\text{g/m}}^{3}$, and a median shifted toward 20 $\mu {\text{g/m}}^{3}$. The proportion of outliers is relatively low (1.2%), suggesting greater variability and a stronger influence of topographic factors compared to the lowland-dominated Cluster 0.
3. **Central Transitional Zone (Clusters 3 and 6)**: This central zone includes parts of Mazovia, Łód ź, and Kuyavia, and is subdivided by topographic and hydrological characteristics. We propose to divide it into two subzones:
  - **Upland-Lowland Transition Zone within Cluster 3.** This cluster is characterized by a boxplot ranging from 20 $\mu {\text{g/m}}^{3}$ to 40 $\mu {\text{g/m}}^{3}$, with the median shifted toward lower values. The maximum concentration reaches 399 $\mu {\text{g/m}}^{3}$, and the proportion of outliers is 2.2%. This region encompasses the transitional belt between central uplands and northern lowlands, including areas such as the Przedbórz Upland, the South Masurian Upland and adjacent plains. The clustering pattern suggests that *PM*_10_ dynamics here are shaped by the interplay of elevation gradients, orographic influences, and regional air circulation.
  - **Lake and River District Zone within Cluster 6.** This cluster also represents a transitional area; however, due to its geographic location, it clearly differs in terms of average concentration values. The box width is comparable to that of Cluster 3, but it is shifted toward lower values. The maximum concentrations are also lower, which may be associated with a stronger influence of unrestricted airflow dynamics. Spanning the river valleys of central Poland—such as the Noteć basin—and extending into the Pojezierze lake districts, this macroregion is shaped by terrain depressions and hydrological features. These local geographical and meteorological conditions appear to modulate pollutant dispersion and retention, contributing to the distinctive *PM*_10_ cluster.
4. **Urban-Industrial Southern (Clusters 1, 4 and 5)**: Concentrated in the Upper Silesian Industrial Area and urban parts of Lesser Poland, this macroregion includes heavily urbanized and industrialized areas such as Katowice, Kraków, Rybnik, and Zabrze. These clusters reflect localized emission sources and complex urban topography, leading to higher and more variable *PM*_10_ concentrations. Clusters 1, 4, and 5 exhibit very similar characteristics, with Cluster 4 standing out due to significantly poorer air quality indicators. It has the highest proportion of outliers—over 5.8%—and extremely high maximum concentrations, reaching nearly 510 $\mu {\text{g/m}}^{3}$. This cluster is located in the industrial region of Poland, adjacent to the Upper Silesian Industrial Area, where both anthropogenic activity and local topography contribute to elevated values. Clusters 1 and 5 also show substantially high maximum concentrations, exceeding 300 $\mu {\text{g/m}}^{3}$.

A comparable approach was adopted in a study on *PM*_2.5_ pollution in China [41]. China, much like Poland, has a highly diverse topography, which makes it a valuable case for comparison. In their analysis, the authors employed a combination of frequent itemset mining and agglomerative hierarchical clustering to identify recurrent patterns of air pollution and to delineate groups of regions with shared characteristics. This procedure resulted in 13 clusters, which were subsequently consolidated into three broader divisions. Each division was distinguished by a unique configuration of climate, degree of urbanization, topography and air pollution patterns. On the basis of these findings, the authors concluded that air pollution management and policy design should move beyond administrative boundaries and instead focus on regions defined by common pollution profiles. This perspective is closely aligned with the rationale of the present study.

These macroregional divisions highlight the strong spatial heterogeneity of daily *PM*_10_ concentrations in Poland and emphasize the influence of both physiographic and anthropogenic factors. Importantly, such spatial structure is markedly less pronounced in annual-mean data, underscoring the value of high-resolution temporal analyses. Furthermore, discrepancies observed between daily and annual cluster assignments—for example, in the Kraków (Bujaka vs. Bulwarowa) or Silesian region (Zabrze vs. Rybnik)—suggest that local microclimatic conditions and proximity to small water bodies may significantly affect *PM*_10_ variability. These findings align with previous studies suggesting that water vapor can act as a precursor in particulate matter formation, particularly in the presence of stagnant atmospheric conditions.

Spatiotemporal analysis for 16 clusters of *PM*_10_ concentrations does not confirm the initial hypothesis that regional policies at the voivodeship level influence the formation of clusters. However, this does not imply that such policies have no impact on pollution levels. The case of Kraków demonstrates that implementing a policy limited to a single subregion does not lead to a significant overall improvement, due to the inflow of pollutants from neighboring areas [15]. This phenomenon is largely driven by the local topography. This observation is further supported by a study on *PM*_2.5_ in China [41], which found that air pollution clusters do not correspond to administrative boundaries or the policies associated with them. The authors highlight that, due to the transboundary transport of *PM*_2.5_, effective mitigation requires coordinated control measures across multiple administrative regions. Consequently, regions defined based on air pollution data extend beyond administrative boundaries and are largely independent of them. Together, these findings reinforce the conclusion from our analysis of *PM*_10_ clusters: policies limited to a single administrative unit may be insufficient to control air pollution effectively. In the data segmented into 16 clusters, it is evident that in the southern, mountainous regions of Poland particularly in Silesia and Lesser Poland - there is a tendency for isolated clusters of daily averages to form. This is likely linked to local accumulation in topographical depressions. In contrast, in the northern lowlands, the situation appears to be much more stable.

Annual average data show cluster transitions, but the overall spatial stability of the groupings remains high. Due to the limited number of spatial locations with *PM*_2.5_ data, the high-resolution spatial macro-analysis did not include these results for macroregion delineation, both for k = 7 and k = 16. An interesting observation from the *PM*_2.5_ dataset is that annual averaging shows greater spatial variability compared to *PM*_10_ possibly due to differences in particle weight, transport mechanisms, and the formation of secondary pollutants in the atmosphere. However, a similar north-south division, as observed with *PM*_10_ is still noticeable. For 16 clusters, the conclusions are analogous: Silesia remains strongly isolated, while Lesser Poland shows significant spatial granularity.

A particularly intriguing result arises from the clustering of daily data and *PM*_2.5_/*PM*_10_ ratios—nearly the entire country falls into a single cluster, with the exception of a small grouping in the southeast (cluster 4). The remaining clusters consist of isolated individual stations. This suggests that, at high temporal resolution, the origin of particulate matter across Poland is relatively uniform. The differences in concentration appear to be driven less by emission sources and more by transport, removal, and dispersion mechanisms, which affect the spatial density of particulate matter. In the annual perspective with k = 7, there is greater spatial variability: strongly isolated southern clusters and more dispersed northern clusters. This directly reflects the capacity of annual data to reveal dominant trends, such as industrial or natural emissions that may be influenced by the energy consumption [42]. Clustering into 16 regions again shows relatively high heterogeneity across Poland, although naturally lower than in the case of k = 7 clusters. Still, annual averaging reveals increased spatial heterogeneity, with significantly greater differentiation in the south and continued strong distinctiveness of the Silesian voivodeship, along with additional internal diversity within Lesser Poland.

### Future research

Future research will involve a more in-depth analysis incorporating additional features, such as meteorological variables and, where possible, indicators related to human activity, in order to further refine regionalization and the analysis of feature importance, following the methodology proposed in [43]. Subsequently, the results of these studies should be integrated with air quality management and funding allocation plans to enable more effective and rapid improvement of atmospheric conditions through targeted management and resource transfer to areas that most critically require intervention - either because they are highly polluted or are significant sources of particulate matter that disperses to surrounding regions. Regarding transitions between clusters, seasonal studies are planned along with the augmentation of reference stations and low-cost sensor (LCS) networks to more accurately delineate regional boundaries. Such a broad, interdisciplinary, and data-driven approach, combined with social and environmental considerations, will allow for the design of more effective national and local policies and support efforts to protect public health.

### Urban planning and policies

Continuous data collection and analysis represent essential components in the pursuit of improved air quality in Poland. The application of machine learning techniques enhances analytical capabilities and facilitates the identification of underlying patterns in air pollution. Insights derived from such analyses may serve as a foundation for the development of urban planning strategies consistent with the principles of smart city design. Furthermore, local governments could leverage these findings to secure targeted funding aimed at mitigating region-specific air quality challenges. For instance, areas characterized by elevated *PM*_2.5_ emissions resulting from vehicular traffic could prioritize traffic optimization measures to reduce pollution levels, while regions dominated by heavy industry might focus on implementing technologies that limit the dispersion of particulate matter.

A framework for developing nationwide policies. The Polish government is actively implementing new policies and regulatory guidelines aimed at mitigating air pollution. The results of our current and future studies may significantly contribute to the formulation of these documents by ensuring that policy measures are data-driven, evidence-based, and tailored to the specific environmental and socio-economic conditions of individual regions and are in line with the country level source analysis [42]. An alternative approach similar to what was proposed by Zhang et al. [41], involves establishing pollution control zones that transcend administrative boundaries. In the context of Poland, this would entail implementing air quality management measures extending beyond individual voivodeships. Such a system would introduce joint inter-voivodeship control areas, delineated according to macroregions identified through data-driven analysis.

### Limitations

This study has several limitations. Large areas of the investigated region are underrepresented, with southern Poland being characterized by a higher station density compared to the northern part of the country.

Another limitation concerns the presence of substantial data gaps in some stations, occasionally spanning long time periods. These missing data were unevenly distributed both temporally and spatially, which may have influenced the clustering procedure and its subsequent division into macroregions. Although advanced imputation methods were applied to reduce spatial bias, imputation is inherently imperfect and certain relevant information may not have been fully captured in the grouping process.

The study is constrained by the use of the k-means clustering algorithm, which is associated with several methodological limitations [44]. One issue concerns the initialization procedure, as the algorithm begins by randomly selecting the initial positions of the cluster centroids. This stochastic component may introduce variability in the results, depending on the choice of the random state parameter. Furthermore, K-means inherently assumes the presence of compact, hyper-spherical clusters, which limits its effectiveness when applied to datasets exhibiting more complex or irregular shapes.

## Conclusions

This study presents the first large-scale, high-resolution spatiotemporal cluster analysis of *PM*_10_ and *PM*_2.5_ in Poland, based on over 13 million observations from reference-grade monitoring stations over 9 years. By combining absolute concentrations and *PM*_2.5_/*PM*_10_ ratios, it provides novel insights into aerosol behavior across diverse physiogeographic regions. Key findings include:

- Dual-resolution clustering:
  – *k* = 7 was applied to capture broad macroregional differentiation.
  – *k* = 16 was originally intended to explore alignment with administrative boundaries (voivodeships) and local urban-industrial dynamics. However, the resulting clusters largely do not correspond to voivodeship borders, highlighting the dominance of physiographic and emission-driven patterns over administrative divisions.
- Physiographic patterns:
  – Southern mountains (Silesia, Lesser Poland) show high granularity and persistent isolation due to topography and thermal inversions.
  – Northern lowlands form stable, homogeneous clusters shaped by open airflow and lower emissions.
- *PM*_10_ shows clear daily clustering influenced by topography and microclimate, whereas annual means smooth short-term accumulation events.
- *PM*_2.5_ exhibits more homogeneous daily patterns but stronger spatial heterogeneity annually, reflecting secondary aerosol formation and regional chemical regimes.
- Urban-industrial hotspots (Katowice, Kraków, Rybnik) are distinguished by both intensive emissions and geomorphological constraints.
- Lack of systematic alignment with voivodeship boundaries highlights the need for supra-regional coordination, including harmonized standards, joint monitoring, and mitigation strategies.

Based on these results, four macroregions are proposed for PM pollution management in Poland: Western-Baltic Lowland – Carpathian Foothill-Upland, Central Transitional Zone (with sub-divisions), and Urban-Industrial Southern. The framework emphasizes the need for targeted interventions coordinated at a supra-regional level, with maybe centralized allocation of resources from the national level to sub-regions. The current voivodeship-based governance does not align with actual pollution patterns, which may result in inefficiencies in funding and mitigation efforts. A macroregional approach can potentially enable a more effective distribution of financial and technical resources, ensuring interventions are proportional to emission burdens and physiographic constraints, rather than administrative boundaries.

## Supporting information

S1 File (DOCX)
