# Supplementary material for: Air pollution macro-regions identification using machine learning and spatio-temporal analysis
Source: PLoS One. 2026 Jan 12;21(1):e0340191. doi: 10.1371/journal.pone.0340191 (PMC12795359; doi:10.1371/journal.pone.0340191)
Supplement: S1 File — (DOCX) [file pone.0340191.s001.docx]

**Data Availability statement:**

The data underlying the results presented in the study are available from
<https://powietrze.gios.gov.pl/pjp/archives.>
Specifically in folders available for download (first is the link which downloads the folder with all of stated year’s measurements, then are listed specific files within the folder)
<https://powietrze.gios.gov.pl/pjp/archives/downloadFile/236>
- 2015_PM2.5_1g.xlsx
- 2015_PM2.5_24g.xlsx
- 2015_PM10_1g.xlsx
- 2015_PM10_24g.xlsx
<https://powietrze.gios.gov.pl/pjp/archives/downloadFile/602>
- 2016_PM2.5_1g.xlsx
- 2016_PM2.5_24g.xlsx
- 2016_PM10_1g.xlsx
- 2016_PM10_24g.xlsx
<https://powietrze.gios.gov.pl/pjp/archives/downloadFile/262>
- 2017_PM25_1g.xlsx
- 2017_PM25_24g.xlsx
- 2017_PM10_1g.xlsx
- 2017_PM10_24g.xlsx
<https://powietrze.gios.gov.pl/pjp/archives/downloadFile/603>
- 2018_PM25_1g.xlsx
- 2018_PM25_24g.xlsx
- 2018_PM10_1g.xlsx
- 2018_PM10_24g.xlsx
<https://powietrze.gios.gov.pl/pjp/archives/downloadFile/322>
- 2019_PM25_1g.xlsx
- 2019_PM25_24g.xlsx
- 2019_PM10_1g.xlsx
- 2019_PM10_24g.xlsx
<https://powietrze.gios.gov.pl/pjp/archives/downloadFile/424>
- 2020_PM25_1g.xlsx
- 2020_PM25_24g.xlsx
- 2020_PM10_1g.xlsx
- 2020_PM10_24g.xlsx
<https://powietrze.gios.gov.pl/pjp/archives/downloadFile/486>
- 2021_PM25_1g.xlsx
- 2021_PM25_24g.xlsx
- 2021_PM10_1g.xlsx
- 2021_PM10_24g.xlsx
<https://powietrze.gios.gov.pl/pjp/archives/downloadFile/524>
- 2022_PM25_1g.xlsx
- 2022_PM25_24g.xlsx
- 2022_PM10_1g.xlsx
- 2022_PM10_24g.xlsx
<https://powietrze.gios.gov.pl/pjp/archives/downloadFile/564>
- 2023_PM25_1g.xlsx
- 2023_PM25_24g.xlsx
- 2023_PM10_1g.xlsx
- 2023_PM10_24g.xlsx

and <https://powietrze.gios.gov.pl/pjp/archives/downloadFile/622> which are station meta data
